# Flyglow: Single-fly observations of simultaneous molecular and behavioural circadian oscillations in controls and an Alzheimer’s model

**DOI:** 10.1038/srep33759

**Published:** 2016-09-23

**Authors:** Eleonora Khabirova, Ko-Fan Chen, John S. O’Neill, Damian C. Crowther

**Affiliations:** 1 University of Cambridge, Department of Genetics, Downing Site, Cambridge, CB2 3EH, United Kingdom; 2 UCL Institute of Neurology, London WC1N 3BG, United Kingdom; 3 MRC Laboratory of Molecular Biology, Francis Crick Avenue, Cambridge, CB2 0QH, United Kingdom; 4 AstraZeneca, Neuroscience, Sir Aaron Klug Building, Granta Park, Cambridge, CB21 6GH, United Kingdom

## Abstract

Circadian rhythms are essential for health and are frequently disturbed in disease. A full understanding of the causal relationships between behavioural and molecular circadian rhythms requires simultaneous longitudinal observations over time in individual organisms. Current experimental paradigms require the measurement of each rhythm separately across distinct populations of experimental organisms, rendering the comparability of the resulting datasets uncertain. We therefore developed FLYGLOW, an assay using clock gene controlled luciferase expression detected by exquisitely sensitive EM-CCD imaging, to enable simultaneous quantification of parameters including locomotor, sleep consolidation and molecular rhythms in single flies over days/weeks. FLYGLOW combines all the strengths of existing techniques, and also allows powerful multiparametric paired statistics. We found the age-related transition from rhythmicity to arrhythmicity for each parameter occurs unpredictably, with some flies showing loss of one or more rhythms during middle-age. Using single-fly correlation analysis of rhythm robustness and period we demonstrated the independence of the peripheral clock from circadian behaviours in wild type flies as well as in an Alzheimer’s model. FLYGLOW is a useful tool for investigating the deterioration of behavioural and molecular rhythms in ageing and neurodegeneration. This approach may be applied more broadly within behavioural neurogenetics research.

Circadian oscillations underpin the ability of organisms to predict environmental challenges across the day/night cycle, permitting a range of adaptive and pre-emptive biochemical, endocrine and behavioural responses. In humans, aberrant circadian biology is increasingly common with ageing and even more so in neurodegenerative disorders such as Alzheimer’s disease (AD). However there is no consensus as to whether circadian dysfunction is a cause or effect of the neurodegenerative process, consequently the control hierarchies amongst the many circadian oscillators that are present in an organism need further study. These oscillations occur on a number of scales, from the crucial central molecular clock apparatus in the mammalian hypothalamus, through to peripheral molecular clocks in a number of organs and the associated circadian changes observed in hormone levels, metabolic indicators and behaviour, notably the sleep/wake cycle.

At a molecular level the circadian clock machinery is highly conserved from flies, through mice to humans, consisting of a series of interlocked transcription-translation feedback loops observable in every cell^1^. In mammals at least, these rhythms are orchestrated by a central master clock mechanism located within the hypothalamic suprachiasmatic nuclei^2^. The resulting temperature-compensated gene expression rhythms are observed in essentially all tissues^3^, with an endogenous period of approximately 24 hours, and are entrained with the environment by signals such as light, feeding or temperature acting as the entraining cue (*zeitgeber*). Such molecular clock oscillations in *Drosophila* may be visualized non-invasively by expression of firefly luciferase fused with a cycling ‘clock protein’, such as Period. When these organisms are fed luciferin (luciferase substrate), the intensity of the light emitted reports the level of the luciferase fusion protein that in turn faithfully reflects the cycling of the molecular clock in every cell where it is expressed^4^,^5^.

Similarly, circadian behaviours such as locomotor activity and sleep are readily observed in a wide range of animals. In *Drosophila*, sleep episodes are distinguished from wakefulness by periods of locomotor inactivity with decreased sensitivity to arousing stimuli. As in mammals, *Drosophila* sleep is homeostatically regulated and rebounds following deprivation^6^,^7^,^8^. For practical purposes fly sleep is defined as a period of immobility exceeding five minutes^6^,^8^.

There is much interest in mechanistic relationships within the hierarchy of rhythms at cellular, tissue and whole organism levels; for example there is variability in the extent to which peripheral cellular rhythms are slave oscillators, tightly coupled to a master central clock which also co-ordinates behavioural rhythms^2^,^9^,^10^,^11^,^12^,^13^,^14^. Whilst recent technical advances have made it possible to study the relationships between these various oscillations within individual transgenic mice^13^, such approaches are not readily scalable. Higher throughput is possible with invertebrate organisms such as *Drosophila*, and in this case circadian locomotor activity is conventionally monitored by the infra-red beam break based *Drosophila* Activity Monitor (DAM) system^15^. Recent advances in video tracking and the charge-coupled device (CCD) microscopy have increased the precision of *Drosophila* sleep monitoring^16^,^17^ as well as enabling circuit-level recording of molecular clocks *ex vivo*^18^. However, the relationship between molecular and behavioural circadian oscillations must still be inferred by behavioural or molecular observations, sequentially or on different fly populations. Conventional statistical analysis generates parameters such as period, amplitude and rhythm quality with differences between populations being reported largely in terms of variance. By using simultaneous observations of multiple oscillators in single organisms more powerful paired statistical approaches become possible.

To harness this power to understand the relationship between molecular and behavioural oscillations, we have developed a video-tracking locomotor assay to simultaneously record the bioluminescence from peripheral molecular clocks and sleep/activity behaviours in individual *Drosophila*. The characteristics of circadian rhythms in control organisms were compared to those in flies expressing the toxic Aβ_42_ peptide as a model of AD.

## Results

### Simultaneous molecular clock and behavioural measurements: FLYGLOW

**Figure 1:**
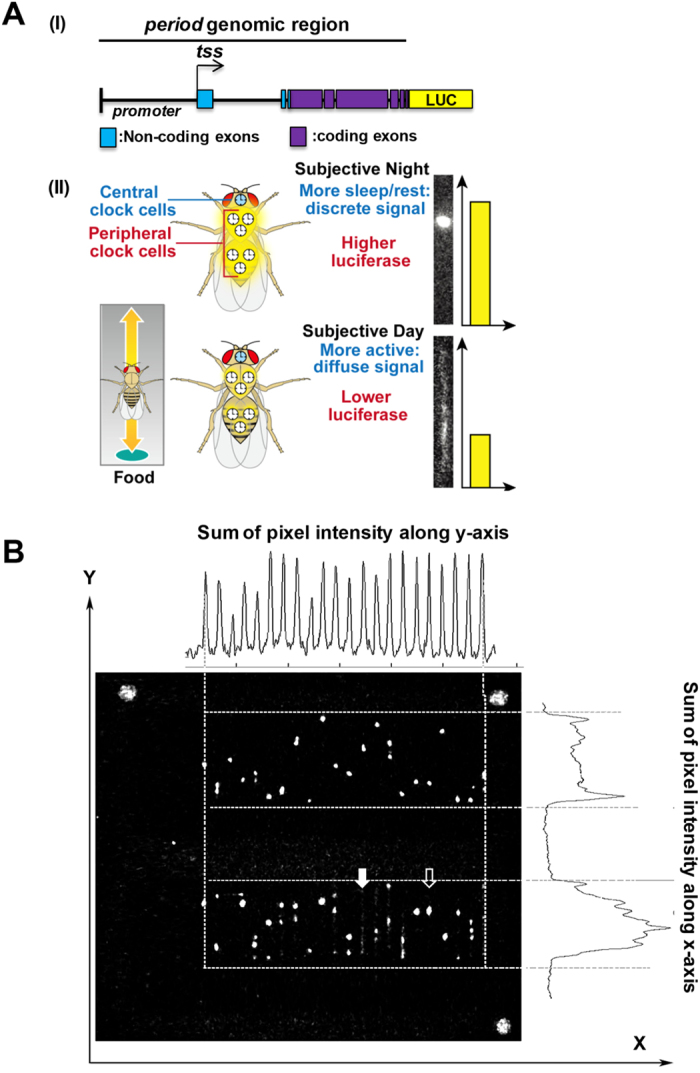
*Period-luciferase*-expressing flies allow simultaneous measurement of the peripheral molecular clock and behavioural phenotypes. (**A**) Panel I: Period-luciferase *XLG-luc2* construct schematic containing period promoter, 5′UTR and full-length period exons and introns. tss: transcription start site. LUC: luciferase. Panel II: Cartoon illustrating control flies generating a higher *Period-luciferase* signal and less locomotor activity during the subjective night, resulting in bright stationary spots in the digital image (upper section). During the subjective day the overall magnitude of bioluminescence is reduced and more evenly distributed along the length of the tube due to movement of the fly (lower section). (**B**) A representative frame from the digital camera. Three bright spots at the corners of the tray were included as landmarks for image orientation. Total pixel intensity projections along the y-axis (upper plot) and the x-axis (right plot) were used to calculate the boundaries of each tube across all the images in an experiment. Active flies (filled arrow) and those at rest (empty arrow) are apparent (see also Fig. 2).

*XLG-luc2* flies (Fig. 1A,I) expressing a Period-Luciferase fusion protein luminesce when fed with luciferin (Fig. 1A,II). This light, detected using a sensitive electron-multiplying CCD camera, reported clock gene expression in peripheral tissues; the signal was lowest at anticipated dawn and highest at anticipated dusk (Figs 1A,II and 3A). When constrained within glass capillary tubes (Fig. 1A) the flies exhibited, essentially one-dimensional locomotor behaviour that was quantified by computational image processing. In these experiments one end of the tube was sealed with fly food containing luciferin and the other end closed with cotton wool; these conditions will support a fly for two weeks. Each frame was produced by integrating luminescence detected for each pixel over sequential 300 sec epochs (Fig. 1B) allowing us to construct a time-lapse movie representing 7 days of fly molecular clock and behaviour (Supplementary movie): for each tube and for each frame there was sufficient information to directly measure the fluctuation in the level of peripheral clock gene expression reported by luciferase activity (number of photons detected *per* tube) as well as locomotor activity (spatial distribution of detected photons within the tube). We could also robustly measure the duration of immobility and so inferred sleep-like episodes according to standard criteria^7^,^16^.

**Figure 2:**
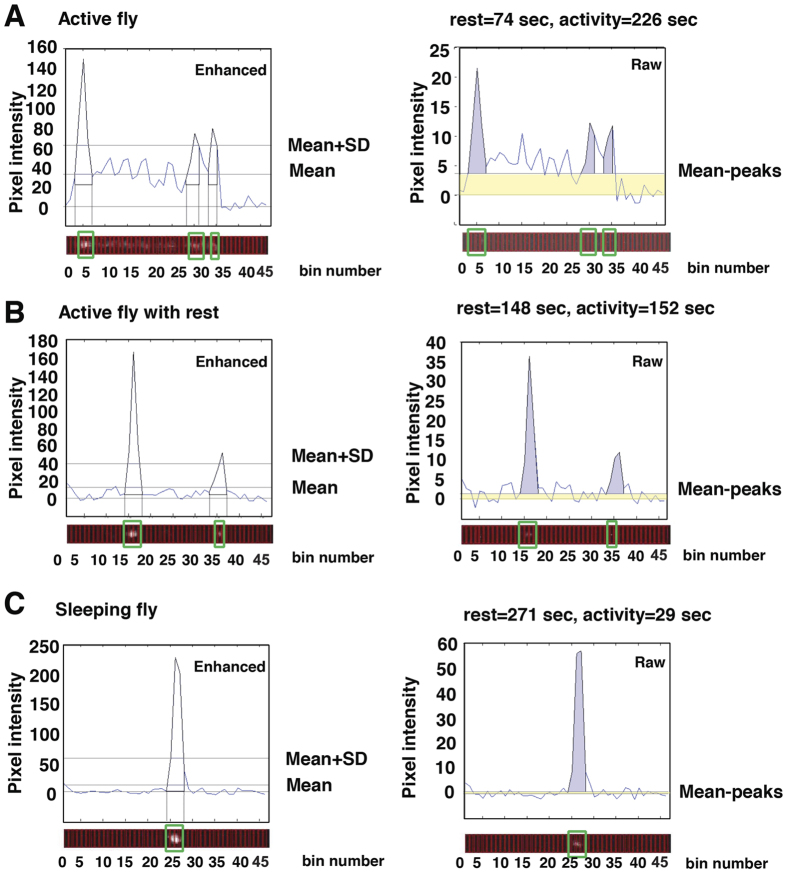
Frame-by-frame measurements of molecular clock and behavioural status. Pixel intensity was plotted and binned into 46 segments along the length of the tube for each image (red boxes on x-axis). The enhanced images (left column) permitted sensitive feature detection and this data used to detect bins containing peaks of intensity in the following way: The mean pixel intensity and standard deviation (SD) for all bins in a tube were calculated. Peaks in the enhanced data were assigned when the intensity of a bin exceeded the mean + SD for that tube. Hereafter only raw intensity data is processed. Using the peak assignments the value of the mean bin intensity, excluding data assigned to peaks (green boxes), was calculated and termed the “Mean-peaks” value. The smear of intensity along the tube, resulting from fly movement, appears as a rectangular area (shaded yellow between Mean-peaks and background) on the intensity profile. The purple area under the peaks represents an episode of rest. The total of purple and yellow areas is equivalent to 300 sec. Representative data is presented for frames containing an active fly with some short rest periods (**A**), a fly exhibiting longer periods of rest (**B**) and a sleeping fly (**C**). The summed pixel intensity within a particular tube indicated the level of the *Period-luciferase* construct and hence the status of the molecular clock.

We found that baseline bioluminescence decreased exponentially with time, probably due to decay of the luciferin. Therefore the data was detrended using the Butterworth low-pass filter with >72 hours threshold so that the processed signal had constant mean and amplitude across the 7-day time course as previously described^19^. The images of the capillary tubes were then processed to detect two patterns of intensity along their length. Active flies appeared as a smear along the tube (Fig. 1A,B, filled arrow) and generated a rectangular intensity profile (Fig. 2, yellow shading). Resting flies generated local bright spots (Fig. 1A,B, empty arrow) that were detected as peaks on the intensity profile (Fig. 2, purple shading). Contrast-enhanced images were used to detect the bright spots of resting flies; thereafter, raw data, processed only to subtract local background camera signal, was used for all quantitative measures. For each 300 sec frame and for each fly our analysis partitioned time according to (i) time spent active, that is the area under the “mean without peaks” (mean-peaks, Fig. 2) rectangle on the intensity profile (yellow) versus (ii) time spent resting, that is the area under the peaks (purple). The sum of all the yellow and purple segments was equivalent to 300 sec for each frame and for each fly.

**Figure 3:**
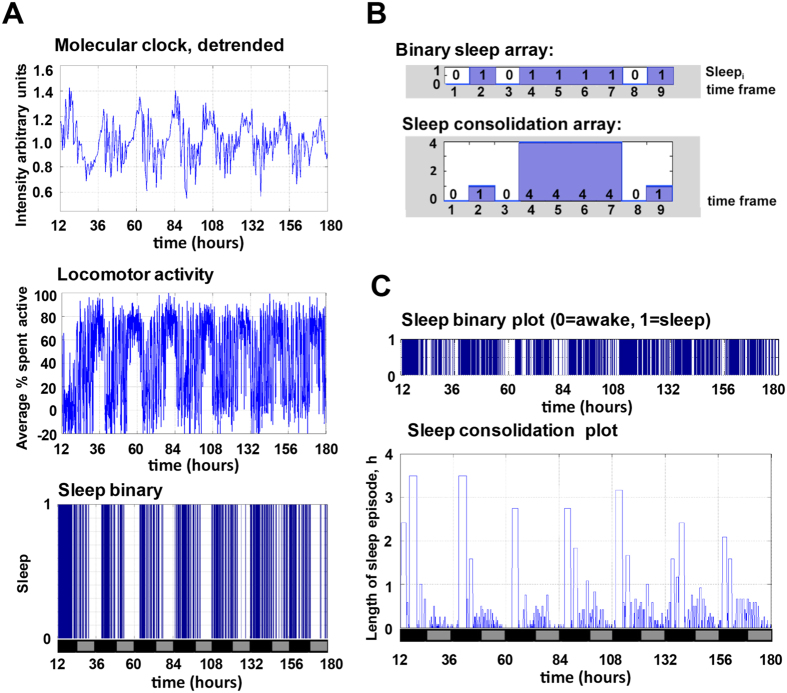
Time course of molecular and behavioural measurements for a single fly. (**A**) The bioluminescence intensity of a representative single fly during seven days of constant darkness reported the oscillation of the molecular clock (Molecular clock). The percentage of time spent active (Locomotor activity) was calculated and the sleep status (Sleep binary) was assigned to a frame as described in the Methods. (**B**) Each frame containing a fly that had been immobile for over 300 sec was assigned a value of 1 in the binary sleep array. All other frames were assigned a value of zero. The sleep consolidation array contained, for each sleep frame, the total length of the encompassing sleep episode. (**C**) This measurement of sleep consolidation exhibited circadian oscillation. X-axis in (**A,C**) indicates circadian time in hours (Time, h) with subjective day (grey) and night (black) indicated. The recording started from subjective dusk (circadian time = 1200 hr).

The peripheral molecular clock signal was then plotted for the population alongside contemporaneous observations of frame-by-frame quantification of activity and sleep (Fig. 3A). The conventional sleep binary plot (Fig. 3A, sleep binary) was determined for each 300 sec frame. Further processing allowed the calculation of fluctuations in sleep episode length (consolidation) (Fig. 3B). The sleep consolidation plot showed that sleep episodes of over 3 hr are commonplace in the early part of the subjective night (marked with black bars on x-axis, Fig. 3C). Indeed, more than 50% of the control flies exhibited at least 1 such long sleep episode in subjective night during the 7 day recording. Later in the night discrete episodes of 40–50 min were seen; by contrast, at least for control flies, there were only shorter naps (<30 min) during the day. This pattern confirms that *Drosophilae* show daily fluctuation in sleep duration^16^,^20^. To further validate that the immobility we detected represented conventional sleep behaviours similar to those detected in both infra-red video tracking and DAM system, we documented sleep position in a separate 7 day recording, and found that majority of the control flies express sleep bouts ~20 mm from the food of the tube (Figure S1), consistent with previous reports^8^,^17^.

### Comparison of FLYGLOW with existing experimental paradigms

**Figure 4:**
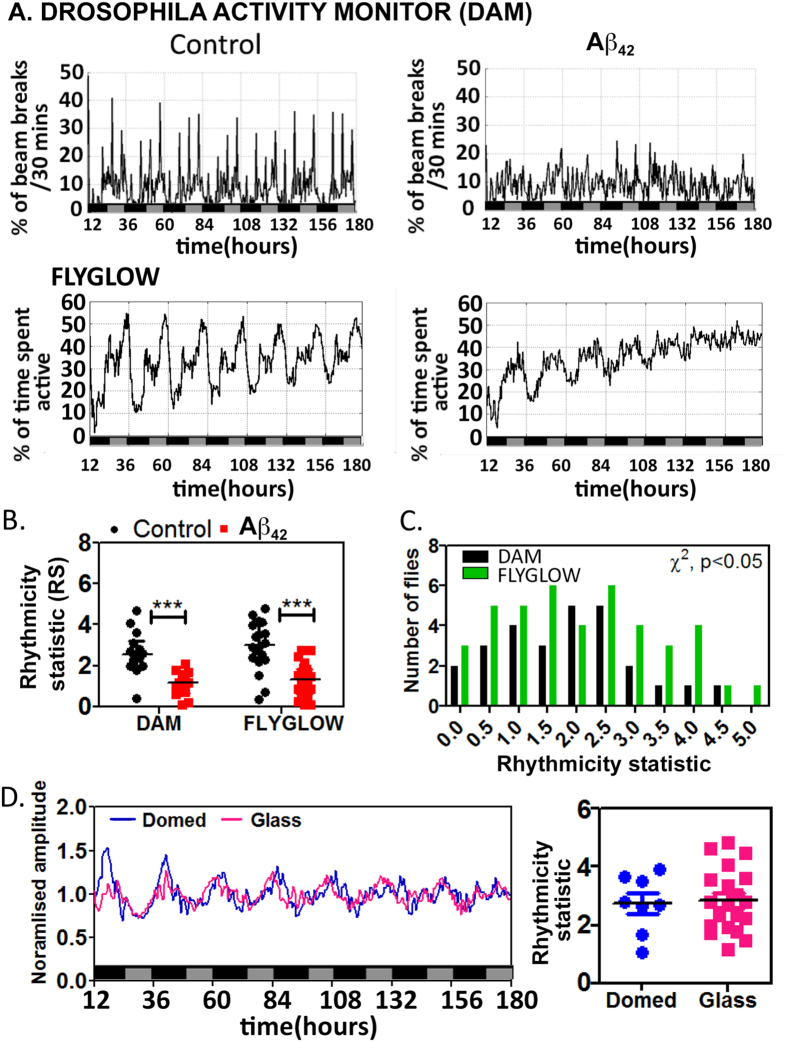
Comparison of FLYGLOW with conventional methods for population measurements of locomotor activity and molecular clock rhythms. (**A**) The mean locomotor activity for control flies (n = 14) and those expressing the Aβ_42_ peptide, as a model of Alzheimer’s disease pathology (n = 13), was assessed using the DAM system (y-axis: percentage of the maximum beam breaks per 30 minutes) and FLYGLOW (n = 20 for control and n = 22 for Aβ_42_-expressing flies). The x-axis represents circadian time in hours (time, h) with subjective day (grey) and night (black) indicated. The recording started from subjective dusk (circadian time = 1200 hr). (**B**) The robustness of locomotor rhythmicity was determined by calculating RS values. Rhythms were significantly stronger in the control flies as compared to Aβ_42_-expressing flies (2-way ANOVA, p < 0.001). The RS value of the FLYGLOW data as a whole was higher than for DAM (p < 0.05, 2-way ANOVA). (**C**) The frequency distributions of RS values for locomotor activity are similar for the two approaches (FLYGLOW n = 42 flies & DAM n = 27 flies) however FLYGLOW is more sensitive to highly rhythmic flies (p < 0.05, χ^2^-test). (**D**) Left panel: Comparing the mean bioluminescence rhythms from FLYGLOW (n = 20, magenta) with flies observed in the classical domed 96-well plates (n = 8, blue) indicates that the molecular clock signals are essentially identical. Right panel: There are no significant differences between RS values of the molecular clock rhythms between FLYGLOW (tube, magenta) and the classical approach (plate, blue) (Student’s t-test).

We then compared the actimetric performance of FLYGLOW with the widely-adopted DAM beam-break-counting apparatus^15^ (TriKinetics Inc., MA USA). We used two populations of flies, the first were strongly rhythmic control flies and the second pan-neuronally expressed the toxic amyloid β peptide (Aβ_42_ pan-neuronally: a model of Alzheimer’s disease, Fig. 4A). We and others have recently shown that Aβ_42_-expressing flies showed progressive locomotor dysrhythmias as they age despite their central molecular clock remaining essentially intact^21^,^22^. Moreover, significant sleep disruption was recently reported in Aβ_42_-expressing flies^23^. Therefore, we decided to use Aβ_42_-expressing flies as our positive control for detecting detailed circadian behaviour changes. Visually, the average locomotor profile for the 23 day old control flies (left panels) shows relatively higher amplitude rhythmic signals in FLYGLOW as compared to the DAM system, likely due to the fact that FLYGLOW measures not activity but time spent being active, which reduces sharp burst peaks (DAM, Fig. 4A). The Aβ_42_-expressing flies exhibit markedly weaker rhythmicity as compared with controls (Fig. 4A, right panels), appearing essentially arrhythmic in the DAM system but retaining detectable rhythms using FLYGLOW. The quality of each fly’s behavioural rhythmicity may be quantified by calculating a time-delayed auto-correlation statistic (RS value)^19^. In this case we saw that both DAM and FLYGLOW rank Aβ_42_-expressing flies as less rhythmic than controls (p < 0.001 for both techniques, Fig. 4B). The distribution of RS values in the population of flies recorded by FLYGLOW was essentially identical to that in DAM system (χ^2^-test, Fig. 4C), indicating that the new method is at least as sensitive at detecting behavioural rhythms as current methods; it appears however that FLYGLOW may be a more sensitive technique for detecting highly rhythmic flies (RS > 4.0).

Next we compared FLYGLOW molecular clock detection with the performance of the current experimental paradigm in which flies are constrained under small plastic domes within the wells of a 96 well microtiter plate^24^. This comparison was undertaken with the goal of excluding the possibility that the extra freedom of movement in the FLYGLOW apparatus could add a systematic artefact to the system: for example the availability of the luciferin substrate might be less in the tubes and result in artefactual changes in bioluminescence. As shown in Fig. 4D, the two approaches generated essentially identical bioluminescence traces; moreover the fly-by-fly rhythmicity analysis indicated that the RS values were also very similar.

### FLYGLOW simultaneous comparison of molecular and behavioural oscillations

**Figure 5:**
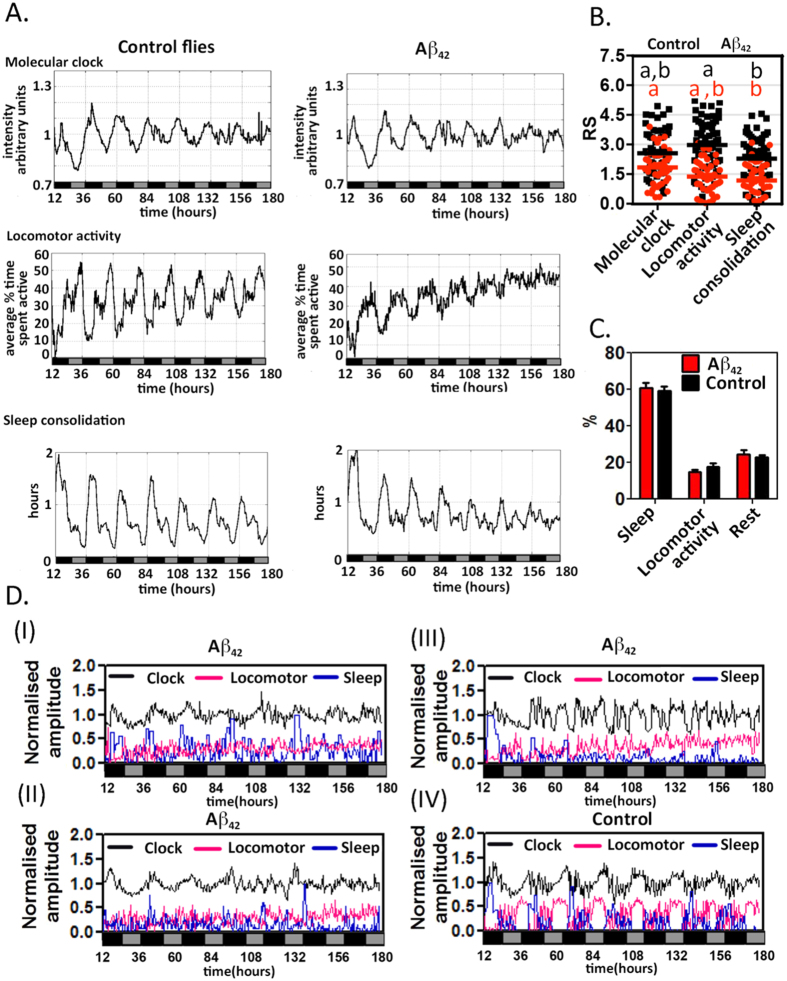
Comparing the rhythmicity of control and Aβ_42_-expressing flies. (**A**) The representative population means of *XLG-luc2* signal intensity (Molecular clock), percentage of time spent active (Locomotor activity) and sleep episode length (Sleep consolidation) were plotted for control (n = 60), and Aβ_42_-expressing (n = 45), flies. (**B**) All three rhythms (Locomotor activity and Sleep consolidation) were significantly less robust in Aβ_42_-expressing flies (Aβ_42_, red) as compared to controls (Control, black) as determined by comparison of RS values by one-way ANOVA and indicated by the colour lettering. Repeated measures one-way ANOVA was used to identify statistical differences in RS values for various pairs of rhythms within each genotype. The same letter label indicates *no* difference (p > 0.05) within each genotype. (**C**) Despite the loss of rhythmicity seen for Aβ_42_-expressing flies, there were no significant differences in the relative partitioning of time between sleep, locomotor activity and rest (χ^2^-test or matched 2-way ANOVA). (**D**) A variety of circadian phenotypes are observed in single fly recordings: Panels I & II: two traces of Aβ_42_-expressing flies with weak behavioural rhythms (locomotor activity in magenta and sleep consolidation in blue) in panel I the molecular clock (black) remains robust whereas in panel II the clock is lost after day 4. Panel III: Aβ_42_-expressing fly with all signals non-rhythmic. Panel IV: representative control fly with robust molecular and behavioural rhythms. Locomotor activity and sleep consolidation amplitudes are normalized to the longest activity and sleep episode respectively. X-axis in (**A,D**) indicates circadian time in hours with subjective day (grey) and night (black) indicated. The recording started from subjective dusk (circadian time = 1200 hr).

Three principal datasets are generated by the FLYGLOW system: (i) the oscillation of the molecular clock in peripheral tissues as reported by total bioluminescence, (ii) percent of time spent in locomotor activity and (iii) sleep consolidation as estimated by average sleep episode duration. To allow comparison with existing approaches these parameters were calculated for populations of control and Aβ_42_-expressing flies (Fig. 5A). Control flies aged 23–30 days (left column, three biological repeats, n = 60 flies total) showed high amplitude molecular oscillations, with gradual dampening,^25^ that were most intense in the early part of the subjective night. Whereas in flies expressing the Aβ_42_ peptide (right column, three biological repeats, n = 45 flies total) the rhythms were significantly less robust, particularly from day 4 onward (Fig. 5A,B).

For control flies, locomotor activity was also highly circadian, exhibiting two peaks: the first occurring just before anticipated, and subjective, dusk, when flies spent up to 50% of their time moving, and the second just before subjective dawn when they were moving approximately 40% of the time. The locomotor behaviour for Aβ_42_-expressing flies exhibited a low-amplitude, less regular rhythm was characterized as a state of continuous restlessness throughout the subjective day and night.

As expected, the sleep consolidation plot was highest when the locomotor activity was least. Specifically, the duration of the average sleep episode in the early night, when control flies spent <10% of their time moving, was 80–100 min (Fig. 5A, left panels). During the morning of the subjective day the flies took short naps of up to 30–40 min at a time when they exhibited significantly less locomotor activity (20% time spent moving). Sleep episodes were at their shortest during the late afternoon peak in locomotor activity.

Qualitatively the rhythms observed in Aβ_42_-expressing flies were less robust than controls (Fig. 5A) and this was confirmed by calculating auto-correlation rhythmicity statistic (RS) values (black vs. red lettering Fig. 5B). A higher percentage of control flies showed rhythmic (RS >1.5) profiles (86% for molecular oscillations, 85% for locomotor activity, and 71% for sleep consolidation) as compared to Aβ_42_-expressing flies (66% for molecular oscillations, 42% for locomotor activity and 42% for sleep consolidation). Furthermore as compared to control, the overall sleep structure is altered, with Aβ_42_-expressing flies showing short, fragmented sleep bouts in the subjective night and longer compensatory naps in the subjective day (Figure S2), a feature that echoes the daytime somnolence in AD. Remarkably these changes in rhythmicity did not cause any overall difference in the relative proportions of sleep (sum of rests longer than 5 min), activity and rest (sum of rests shorter than 5 min) (Fig. 5C). Thus the differences in sleep related entirely to its temporal organization and not its total amount.

The plotting of representative data from individual flies was particularly instructive (Fig. 5D). For example many Aβ_42_ flies exhibited weak behavioural rhythms alongside more robust molecular rhythms (panel I). However in this context, it was not uncommon to see age-related loss of even the molecular clock (panel II, day 4 onwards). At one extreme an individual Aβ_42_ fly may never show rhythmicity in any of the three signals (panel III) while the majority of control flies remained entirely rhythmic throughout the week of recording (panel IV). Importantly behaviourally rhythmic flies with chaotic molecular signals were not observed.

### Dissecting the mechanistic links between the various oscillators

**Figure 6:**
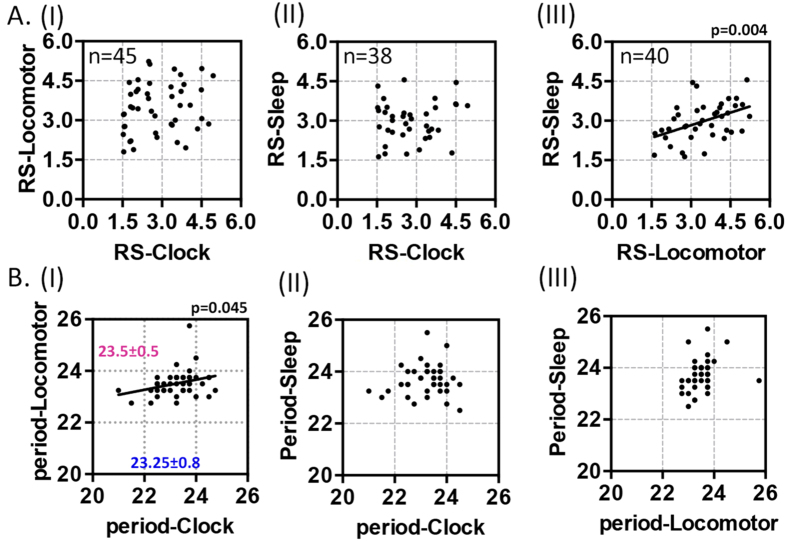
RS and period correlation analysis: dissecting causal relationships. (**A**) The paired RS values for the various rhythms were plotted for each fly. There was no correlation between RS values for molecular clock (RS-Clock) and locomotor activity (RS-Locomotor, panel I) or sleep consolidation (RS-Sleep, panel II). By contrast the RS values for locomotor activity (RS-Locomotor) and sleep consolidation (RS-Sleep) rhythms where correlated (panel III, p = 0.004). Flies were only included in this analysis if they were rhythmic (RS > 1.5) for both tested signals; consequently the n-values vary between panels. The significance of the RS correlations was determined by the *Pearson r*. Only control (non-Aβ_42_) flies were tested. (**B**) The paired signal periods for the various rhythms were plotted for each fly. As above only rhythmic flies were considered. The periods of the locomotor activity (period-Locomotor) were correlated with the periods of the molecular clock (period-Clock, panel I, p = 0.045). By contrast there was no correlation between the periods of the sleep consolidation (period-Sleep) and either circadian rhythm (panels II & III). The average periods of the locomotor activity rhythms are longer than those of the molecular clock (panel I, paired t-test, p < 0.05). The significance of the correlation between periods was determined by the *Pearson r*.

Our single fly measurements permit powerful analysis of correlations between the robustness and the period of the oscillations in the three datasets. Furthermore the simultaneous observation of multiple signals within a single fly allows the use of paired statistical tests, something that is not possible when comparing separate population-based measurements. To explore such advantages, our further analysis focused on the control flies (Fig. 6). Considering rhythm robustness, it is widely accepted that if the RS values of two oscillations are correlated then the signal generators are likely to be coupled. For example when one of the rhythms is robust in a particular fly one expects that the coupled rhythm will be similarly robust, and vice-versa. Our three-way analysis of RS correlations in control flies showed that there is no evidence for coupling between the peripheral molecular clock and either of the two behavioural phenotypes (Fig. 6A, panel I: RS-Clock vs. RS-Locomotor & panel II: RS-Clock vs. RS-Sleep). The RS values for sleep consolidation and locomotor activity were found to be correlated (panel III: RS-Sleep vs. RS-Locomotor, p = 0.004). The inference is that these oscillations are coupled.

A similar three-way comparison of rhythm periods in individual flies, revealed correlations between locomotor activity and the peripheral molecular clock (Fig. 6B, panel I: period-Locomotor vs. period-Clock, p = 0.045). When the periods of oscillation are correlated, even in the absence of direct coupling, it is likely that the underpinning molecular mechanisms are shared. This is because molecular factors that modify the period of one rhythm, within a particular organism, may result in covariance in the period of the correlated rhythm. By contrast, the periods of sleep rhythms do not correlate with those of peripheral clock or locomotor activity (Fig. 6B, panels II & III), indicating that sleep rhythms may be influenced by additional mechanisms such as homeostatic regulation^26^,^27^.

## Discussion

Not only do circadian rhythms have profound roles to play in biology, they are also recognized as important factors in human disease. On one hand environmental disruption of circadian rhythms increases the risk of, for example, obesity and diabetes while on the other hand some diseases are accompanied by circadian deficits from an early stage^28^,^29^. Neurodegenerative disorders, not least AD are characterized by sleep-wake abnormalities, indeed it is night-time confusion and wandering that often precipitates the need for institutional care^30^. Considering a recent finding that any use of (benzodiazepine) sleeping tablets is linked to a 50% increase in the risk of a diagnosis of AD, it is apparent that circadian sleep deficits become symptomatic early, as part of a dementia prodrome^31^. As AD progresses, patients will exhibit marked sleep fragmentation and loss of the normal day/night partitioning of sleep. This may result in a clinically significant state of confusion and agitation, worse in the early evening, termed sundowning^32^,^33^,^34^.

*Drosophila melanogaster* is a model organism that phenocopies key aspects of the deterioration in circadian/sleep organization associated with human ageing and neurodegeneration^21^,^22^,^23^,^35^,^36^,^37^,^38^. However existing experimental paradigms do not allow the longitudinal monitoring of multiple behavioural and molecular markers in single animals.

We have therefore developed FLYGLOW to investigate the causal relationships between molecular clock oscillations and behavioural and sleep rhythms, in health and an AD model. We make use of a fly strain expressing a clock gene::luciferase reporter construct in peripheral tissues. Furthermore, we incorporate the luciferase substrate luciferin in the fly food at concentrations sufficient to sustain bioluminescence during sleep (when flies do not eat).

Using an ultra-sensitive camera, with exposure times that match the current working definition of a minimal fly sleep episode, we have analysed the frame-by-frame distribution of light emitted by flies, allowing a robust estimate of behaviour. In this analysis locomotor activity appears as a smear of light along the tube and a rest as a bright spot. A sleep episode is defined as two or more frames containing a single resting peak that remains at the same position throughout. The duration of the sleep (defined as >300 sec) is the sum of all the time assigned to the peak summed across >2 frames. It is conceivable that this definition may include some rare events whereby the fly is not actually immobile throughout, but moves within each frame and comes to rest again at the same position frame after frame. That this scenario is unlikely is clear when we consider a time-resolved heat map of sleep positions across the experiment (Figure S1). The first conclusion is that sleep episodes are assigned, as expected, most commonly near the food end of the tube^7^,^16^; the second observation is that the spatial distribution of this sleep preference is wide enough such that flies would be very unlikely to return frame after frame to the same position.

There are however some limitations to the FLYGLOW approach, For example, other infra -red light video tracking may provide greater resolution to allow the detection of complex behaviours, such as grooming^16^,^17^, which are undetectable in our assays since signal detection is limited by the anatomical distribution of luciferase expression (largely in head, thorax and abdomen)^24^. In addition, FLYGLOW does not allow experiments under light-dark cycles; however the system has potential applications in the analysis of non-photic entrainment, for example in response to thermal or proprioceptive stimuli^39^. Taken together the system permits the power of monitoring an array of 50 individual organisms, allowing us to assign simultaneous molecular, locomotor and sleep events over a 7-day period of darkness.

Our initial observation was that, as a population, flies show complex patterns of sleep and locomotor behaviour that remain synchronized in constant dark. Sleep consolidation reached remarkably high levels in the early night, with episodes of 2–3 hours being commonplace. In the flies expressing the Aβ_42_ peptide these long episodes were truncated and instead sleep was partitioned more uniformly across the day, resulting in longer day time naps and shorter episodes of night time sleep (Figure S2). Importantly we did not detect any overall change in the relative amounts of rest, sleep and activity in the control and Aβ_42_-expressing flies indicating a change in the structure rather than the amount of sleep between the two groups (Fig. 5 and Figure S2). These changes in sleep structure strikingly resemble the deficits seen in patients with AD^30^,^32^. The night time sleep shortening in Aβ-expressing flies is consistent with a recent report using the DAM system^23^, suggesting our system is suitable for detecting subtle sleep changes. However we did not detect subjective daytime sleep reduction, probably reflecting the different driver (neuronal vs. global) and the light regimes (constant darkness vs. light-dark cycles) in our recording system as compared to those in Tabuchi *et al*.^23^.

Whereas we detected weaker peripheral molecular rhythms in Aβ_42_-expressing flies as compared to controls at the population level (Fig. 5A and black vs., red lettering Fig. 5B), our simultaneous observation of molecular and behavioural rhythms revealed that the molecular clock in peripheral tissues was more robust than its behavioural counterparts in Aβ_42_-expressing flies (red letters, Fig. 5B). This echoes a number of studies in which the central molecular clock becomes uncoupled from behaviour in Aβ_42_-expressing flies^21^,^22^. This study also revealed many examples of flies exhibiting robust molecular oscillations contemporaneously with behavioural arrhythmia, indicating that our simultaneous recording approach better describes the process of circadian decay as compared with conventional population comparisons. This recurring observation of molecular-behavioural dissociation suggests that one approach to re-entraining disturbed behavioural rhythms may be to enhance the output of the central clock.

Our approach also permits single-fly estimates of RS and period correlations between the molecular and behavioural phenotypes, allowing dissection of their causal relationships. In this respect, we observed no correlation between the RS values for the peripheral molecular clock and either of the behavioural rhythms. This finding confirms the absence of coupling between the global peripheral molecular clock and fly behaviour, as suggested previously by a number of investigators^9^,^10^,^40^. The presence of period correlation between rhythms, as seen for the peripheral molecular clock and locomotor activity allows us to infer that they are underpinned by the same molecular mechanisms. Demonstrating this sort of relationship is not possible using existing population studies. Similarly our approach may be useful for investigating the emerging role of the peripheral clock in the process of ageing^35^,^36^,^41^ as various aspects of circadian rhythms progressively degenerate^42^.

In the FLYGLOW system, sleep is not the simple reciprocal of locomotor activity for two reasons: firstly there is a third behavioural component, namely rest (inactivity lasting <300 sec), which contributes to the daily time partition; secondly, sleep consolidation is a further derivative of sleep, representing the fluctuation of length of sleep episodes. However the correlation between the RS values for sleep consolidation and locomotor behaviour indicates that these rhythms are indeed coupled; the intriguing absence of a period correlation between sleep and the other circadian rhythms points to more complex regulation of sleep/wake that may respond to other homeostatic and regulatory signals^7^,^43^,^44^. Similar dissociation occurs in humans during forced desynchrony (14 hr:14 hr, light:dark day) when circadian physiology becomes decoupled from sleep-wake cycles.

In conclusion, we have developed a broadly applicable approach that allows the causal relationships between molecular and behavioural circadian rhythms to be dissected by simultaneous molecular and phenotypic observations in individual flies. We have combined the current molecular and behavioural assays to allow simultaneous observations in individual organisms. In particular this allows us to use paired statistical tests to understand the correlations between the robustness and period of oscillations and so infer the causal relationships between signals.

## Materials and Methods

### Fly strains and husbandry

All *Drosophila* strains in this study were housed and aged on standard cornmeal food. Flies expressing the E22G (Arctic) variant of amyloid beta peptide 1–42 (Aβ_42_) were used as a model of amyloid toxicity and are described elsewhere^22^,^45^. To monitor clock gene expression in control or pan-neuronally Aβ_42_ expressing flies, a new fly strain (*elav-gal4;; XLG-luc2*/*TM3*) containing *elav-gal4*^*c155*^driver and the *period* promoter driven Period-luciferase fusion construct, *XLG-luc2* (Fig. 1A)^25^, were generated and crossed to the *UAS-A*β_*42*_ or a background control strain. The following offspring were studied: *elav-gal4;uas-Aβ*_*42*_/+*; XLG-luc2*/+ and *elav-gal4;; XLG-luc2*/+. The control and UAS-Aβ_42_ flies share the same *w*^*1118*^ background and both contain *attB* sites derived from phiC31 mediated transformation^22^.

### Fly arena

Individual luciferase expressing flies of required genotypes and ages were housed in a capped glass tube (cap: CAP5-Black; capillary: 5 mm × 65 mm, PGT5 × 65, Trikinetics Inc. USA) containing 100 μl of 1% w/v agar, 5% w/v sucrose and 15 mM (run 1, comparable with ref. 24) or 50 mM (run 2 and 3) luciferin. These tubes were placed in a microscope slide storage tray (100 slide model, 165 mm × 210 mm × 35 mm, Fisher) with card dividers between the tubes (8 mm × 72 mm, 480 GSM, Ryman Limited, UK) (Supplementary Figure 1A,C). Each tray holds up to 48 tubes. A customized arena with the mentioned dimension was constructed using black nylon plastics with fixed spacers (Figure S3, distributed by Polygonal Tree, London, http://polygonaltree.co.uk/).

### Bioluminescence recording

Flies, 20 days post eclosion, were placed in capillary tubes and exposed to a 12-hr light: 12-hr dark (LD) regimen for three days of circadian entrainment prior to being transferred into recording conditions at anticipated dusk (ZT12). Recordings were performed under constant darkness at 26 °C over seven days. Bioluminescence was detected using an EM-CCD camera (Hamamatsu Photonics UK Ltd, C9100-14) cooled to −70 °C incorporated within a Cairn Alligator system (Cairn Research Limited, UK, which is itself within a temperature controlled dark room (Figure S3). A bright field image was taken before each recording to ensure appropriate focus and tray alignment (Figure S3C). Bioluminescence images were recorded with contiguous 5 min integrations over 7 days with camera settings: 4x gain, 200x EM gain (Fig. 1B) that are within the range of linear sensitivity. An example bioluminescence time series is included as a compressed movie, each frame resulting from a 5 min exposure (Supplementary video). Three, week-long recordings were made with trays containing a total of 60 control and 45 Aβ_42_-expressing flies. In separate experiments, control flies were tested for sleep position analysis (50 mM luciferin, Figure S1) or loaded individually into the wells of a microtiter plate containing the food-luciferin substrate (15 mM luciferin) where their movement was restricted by covering, pierced plastic domes^24^. The plate was placed in the Cairn Alligator system and recorded in parallel with the tube-based assay condition. The behaviour of equivalent flies was also recorded using the DAM system beam-breaking apparatus^15^ (TriKinetics Inc., MA USA).

### Image rotation, background subtraction and feature enhancement

The raw data from the camera consisted of the 16 bit photon counts summed over 300 sec at a 1024 × 1024 pixel resolution. Pre-processing of the data began with an estimation of the intensity of the background signal. This was achieved by averaging the brightness of pixels in a 64 × 64 square at the bottom right of the screen; this average value was then subtracted from all pixel intensities. The next step was the rotation of each frame so that the fly tubes become properly aligned with their long axes parallel to the y-axis of the image. This was achieved by manual location of three marker points (grey dots in corners, Fig. 1B) on the tray in which the fly tubes are placed. Once correctly aligned the corresponding tubes in successive rows lie directly above each other on the y-axis. In order to identify where, across the image, each column of tubes was located we plotted the profile of the pixel intensities summed along the y-axis. This profile oscillates, with each peak of the summed pixel intensities indicating the centre of each tube (Fig. 1B). Likewise, the pixel intensities were summed across the image to detect the top and bottom of each row of tubes. A boundary rectangle was then assigned to each tube, assuming that each tube was 17 pixels wide.

Camera vignette effects resulted in small, systematic variations in pixel brightness across the image. To correct for such artefacts a process of tube-by-tube background subtraction was undertaken by first estimating the intensity of each tube’s neighbouring inter-tube dark area. For each tube its particular background rectangle was centred on the midpoint between adjacent tubes, was as long as the tube and was 5 pixels wide. This provided an area sufficient for a robust estimate of background intensity while avoiding contamination with photons from the neighbouring tubes.

At this stage the data was saved as pre-processed “raw data” and was used for all quantitative calculations. To allow sensitive feature detection we also enhanced the contrast and brightness of the images so that flies could be reliably identified despite changes in overall brightness. This process of enhancement used the contrast-stretching transformation algorithm in MATLAB that globally optimizes the dynamic range of pixel intensities. Such processed images were stored as “enhanced data”.

### Detecting resting and moving flies

We then divided each tube rectangle into 4-pixel-high bins along their long axis (y-axis). We summed pixel intensities for each bin and calculated the mean and standard deviation of these values. Using enhanced data, the bins containing a resting fly were identified as peaks in the intensity profile along a tube (Fig. 2, “Enhanced images”); a bin contained a resting fly when the maximum pixel intensity of the peak >(Mean tube intensity + Standard Deviation).

Having identified peaks using the enhanced data, thereafter all analysis was performed on raw data. We then excluded bins that contained resting flies and used the remaining bins to recalculate the mean tube intensity (Mean-peaks, Fig. 2). For each fly and each frame we partitioned the 300 sec of the camera exposure time between two parameters: i) time spent active and ii) time spent resting. The contribution of activity to the profile was measured by calculating the area of the rectangle between the (Mean without peaks) and the background (Fig. 2, yellow areas). The contribution of resting to the profile was measured by calculating the area under each identified peak, bounded by the (Mean without peaks) (Fig. 2, purple areas).

For each frame:


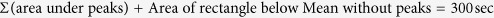



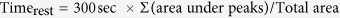



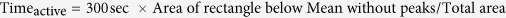


In eight percent of frames, subtraction of the inter-tube background resulted in a negative value for a tube’s mean intensity; this was usually due to artefactual high intensity pixels in a particular inter-tube background region. In six percent of the frames, the brightness of flies was too low to be detected by the camera; in these circumstances the data was discarded and the values of Time_rest_ and Time_active_ interpolated from adjacent time points.

### Measuring the oscillation of the molecular clock

The oscillation of the molecular clock was calculated by summing up the background-adjusted pixel intensity in a rectangle constrained by each tube’s coordinates. This primarily reports cellular circadian rhythms of gene expression in peripheral tissues of the fly^5^.

### Assigning sleep episodes

*Drosophila* sleep episodes are defined as a period of locomotor inactivity lasting for >300 sec. Because of the low photon counts in our experiments, we needed to sum light over a number of seconds. We chose 300 sec as the length of the exposure time for each frame because it provides the ideal resolution for sleep detection. Our analysis of the distribution of photon emission within a tube allows us to detect events, such as rests, with durations substantially shorter than 300 sec. Having calculated the time spent moving and resting for each frame we defined the presence of a sleep episode in the following way: Firstly, a sleep episode can only be initiated in a frame with a single peak of intensity, indicating that the fly was resting in only one position. Secondly, considering the subsequent frames in turn, there must be a peak at the same position, indicating that the fly had rested from one frame to the next. Subsequent frames were analysed sequentially until the resting peak was lost. The component rest times were then summed across the multiple frames; when the total resting time exceeded 300 sec then the whole episode was defined as sleep. This approach is conservative and does not over-identify brief rests as sleep. Each frame within a sleep episode was assigned a 1 in the binary sleep array; all other frames are assigned a zero (1 = asleep and 0 = awake). Using this method, we can robustly partition time into one of three behavioural states for each fly; these are “active”, “resting” and “sleeping” (Fig. 2A–C).

### Calculating sleep consolidation

Our novel sleep consolidation index provides an indication of how long a block of sleep lasts. To calculate this index for a particular fly we looped through the binary sleep array and identified blocks of sleep as consecutive episodes separated by wake periods. Each time point within a particular sleep block was assigned with the duration of its encompassing sleep episode (Fig. 3).

### Final data output

The temporal resolution of the data was reduced to 30 min by binning data from consecutive timepoints, similar to the conventional circadian locomotor assay^22^. For time spent resting or moving, the binned value represented the sum of the constituent timepoints. For sleep consolidation, the binned value was assigned as the mean value of non-zero entries in the sleep consolidation array.

### Time-series analysis

We adapted the well-established autocorrelation methodology to determine rhythmicity and circadian period using Flytoolbox in the MATLab environment. To make our analysis comparable to the DAM actimetry system we have limited our observations to 7-days with data considered in 30 min bins. This constrained paradigm permits the convenient use of Rhythmicity Statistic analysis as described by Levine *et al*.^19^. To normalize the decay of bioluminescence intensity with time we employed the the Butterworth low-pass detrend filter as previously described^19^. Briefly, the trend curve was identified by using low-pass filter that only passes the signal with a frequency lower that 1/72, or with periodicity longer than 72 hours, and attenuates signals with higher frequencies. The bioluminescence signal was then detrended by dividing each data point by the corresponding data point on the trend curve, resulting in a detrended signal with a mean value of 1 and preserving the amplitude of the variation around the trend curve. As a consequence of such filtering, the signal appears to be more robust than in the raw data and the units of measurement are eliminated so that the temporal features can be compared with other signals such as behavioural rhythms. RS values >1.5 was the definition for rhythmicity^46^.

## Additional Information

**How to cite this article**: Khabirova, E. *et al*. Flyglow: Single-fly observations of simultaneous molecular and behavioural circadian oscillations in controls and an Alzheimer’s model. *Sci. Rep.* **6**, 33759; doi: 10.1038/srep33759 (2016).

## Supplementary Material

Supplementary Video

Supplementary Figures
