# Supplementary material for: Flyglow: Single-fly observations of simultaneous molecular and behavioural circadian oscillations in controls and an Alzheimer’s model
Source: Sci Rep. 2016 Sep 23;6:33759. doi: 10.1038/srep33759 (PMC5034315; doi:10.1038/srep33759)
Supplement: Supplementary Figures [file srep33759-s2.pdf]

Supplementary information:

Flyglow: Single-fly observations of simultaneous molecular and behavioural circadian oscillations in controls and an Alzheimer's model

Eleonora Khabirova[1,+], Ko-Fan Chen[1,2,+,*], John S. O'Neill[3*] and Damian C. Crowther[1,4]

[1]University of Cambridge, Department of Genetics, Downing Site, Cambridge, CB2 3EH, United Kingdom
[2]UCL Institute of Neurology, London WC1N 3BG, United Kingdom
[3]MRC Laboratory of Molecular Biology, Francis Crick Avenue, Cambridge, CB2 0QH, United Kingdom
[4]AstraZeneca, Neuroscience, Sir Aaron Klug Building, Granta Park, Cambridge, CB21 6GH, United Kingdom

+ these authors contributed equally
* corresponding authors:
kofan.chen@gmail.com
oneillj@mrc-lmb.cam.ac.uk

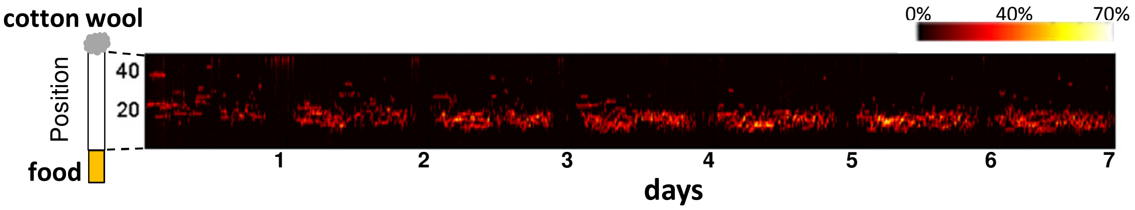

Figure. S1. Time-resolved heat map of population sleep position preference. The position along the tube is presented on the y-axis (bins 1-46) with the food towards the bottom of the plot. Brighter colours represent higher percentages of flies choosing to sleep at a particular position in space and time. The x-axis represents the full seven days of the experiments.

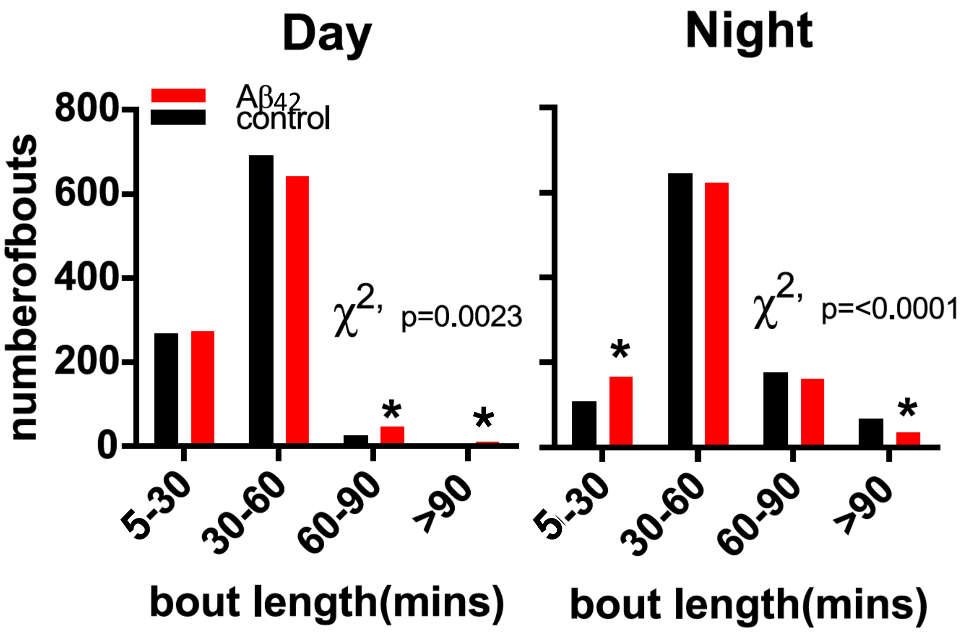

Figure. S2. Distribution of sleep bout lengths. The length of each sleep bouts from Aβ42 expressing flies (n=45) and control (n=60) were calculated throughout the recording and categorised into four groups: 5-30, 30-60, 60-90 and >90 minutes. Whereas Aβ42 expressing flies shifted their subjective day time sleep bouts toward longer length, their night time bouts became shorter as compared to the controls. Asterisks indicates the changes in number of bouts associated with the significant shift in distribution of sleep bout length via X^2 test.

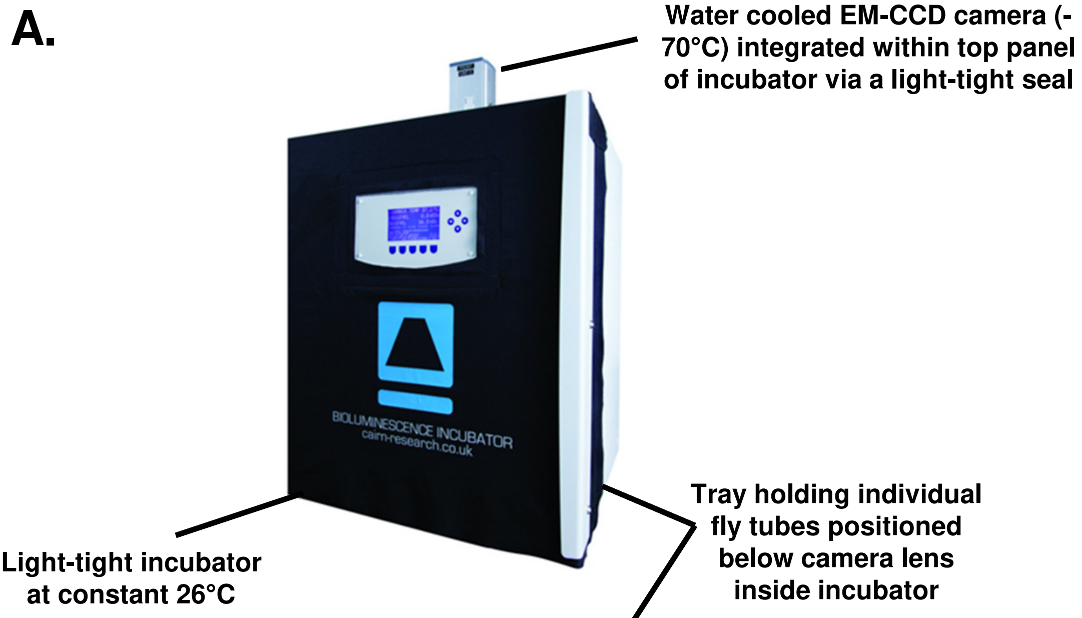

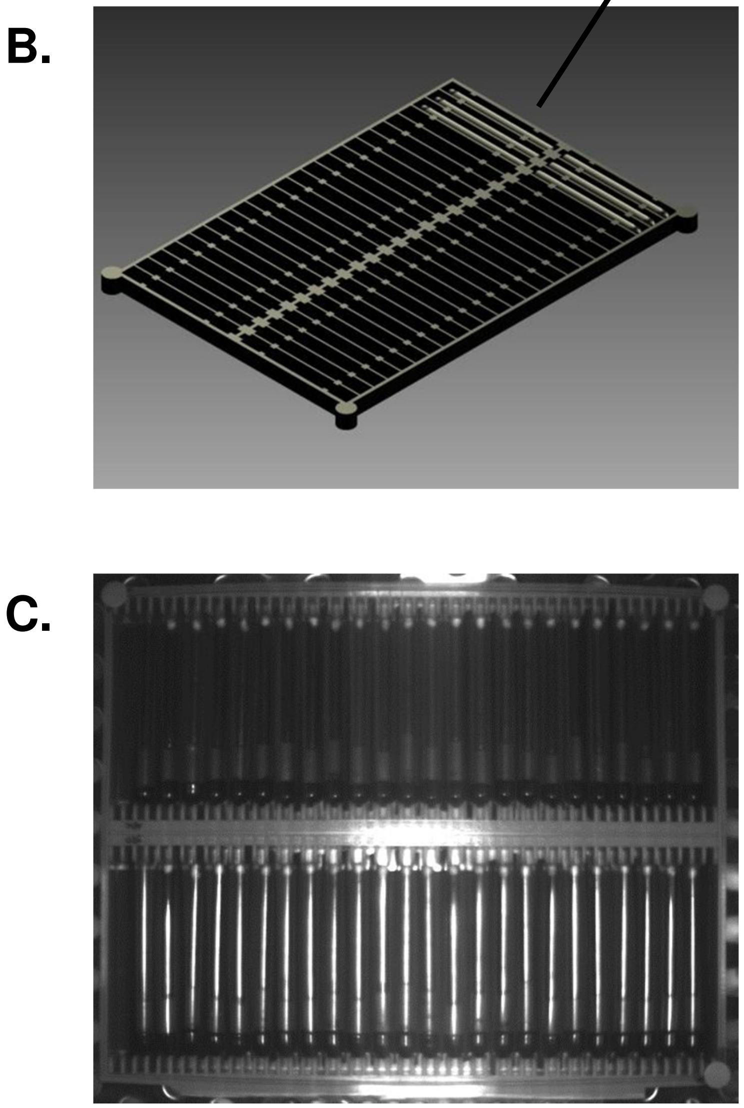

Figure. S3. Instrument setup
(A) An Automated Longitudinal Luciferase Imaging Gas and Temperature Optimised Recorder (ALLIGATOR, Cairn Research) was used for data collection as described in the methods section. The ALLIGATOR was located in a dark room held at constant 20°C.
(B) Illustration of the fly tube arena with the fixed spacer and circular markers
(C) Illustrative bright field image taken by CCD camera to depict field of view. Image acquired with 50 ms exposure under illumination, without EM gain. Three paper circles (grey circles) at the corners of the tray were attached as landmarks for image rotation.
